# Biodegradable and biocompatible subcutaneous implants consisted of pH-sensitive mebendazole-loaded/folic acid-targeted chitosan nanoparticles for murine triple-negative breast cancer treatment

**DOI:** 10.1186/s12951-022-01380-2

**Published:** 2022-03-31

**Authors:** Amirhosein Kefayat, Maryam Hosseini, Fatemeh Ghahremani, Nafise Arbab Jolfaie, Mohammad Rafienia

**Affiliations:** 1 Cancer Prevention Research Center, Department of Oncology, Isfahan University of Medical Sciences, 81746-73461 Isfahan, Iran; 2 Department of Chemistry, Amirkabir University of Technology (Tehran Polytechnic), 1591634311 Tehran, Iran; 3 Department of Medical Physics and Radiotherapy, School of Paramedicine, Arak University of Medical Sciences, Arak, Iran; 4 Biosensor Research Center, Isfahan University of Medical Sciences, Isfahan, Iran

**Keywords:** Mebendazole, Chitosan nanoparticles, Subcutaneous implants, Folic acid, Triple-negative breast cancer

## Abstract

**Background:**

Mebendazole (MBZ) is a well-known anti-parasite drug with significant anti-cancer properties. However, MBZ exhibits low solubility, limited absorption efficacy, extensive first-pass effect, and low bioavailability. Therefore, multiple oral administration of high dose MBZ is required daily for achieving the therapeutic serum level which can cause severe side effects and patients’ non-compliance.

**Method:**

In the present study, MBZ-loaded/folic acid-targeted chitosan nanoparticles (CS-FA-MBZ) were synthesized, characterized, and used to form cylindrical subcutaneous implants for 4T1 triple-negative breast tumor (TNBC) treatment in BALB/c mice. The therapeutic efficacy of the CS-FA-MBZ implants was investigated after subcutaneous implantation in comparison with Control, MBZ (40 mg/kg, oral administration, twice a week for 2 weeks), and CS-FA implants, according to 4T1 tumors’ growth progression, metastasis, and tumor-bearing mice survival time. Also, their biocompatibility was evaluated by blood biochemical analyzes and histopathological investigation of vital organs.

**Results:**

The CS-FA-MBZ implants were completely degraded 15 days after implantation and caused about 73.3%, 49.2%, 57.4% decrease in the mean tumors’ volume in comparison with the Control (1050.5 ± 120.7 mm^3^), MBZ (552.4 ± 76.1 mm^3^), and CS-FA (658.3 ± 88.1 mm^3^) groups, respectively. Average liver metastatic colonies’ number per microscope field at the CS-FA-MBZ group (2.3 ± 0.7) was significantly (*P* < 0.05) lower than the Control (9.6 ± 1.7), MBZ (5.0 ± 1.5), and CS-FA (5.2 ± 1) groups. In addition, the CS-FA-MBZ treated mice exhibited about 52.1%, 27.3%, and 17% more survival days after the cancer cells injection in comparison with the Control, MBZ, and CS-FA groups, respectively. Moreover, the CS-FA-MBZ implants were completely biocompatible based on histopathology and blood biochemical analyzes.

**Conclusion:**

Taking together, CS-FA-MBZ implants were completely biodegradable and biocompatible with high therapeutic efficacy in a murine TNBC model.

**Graphical Abstract:**

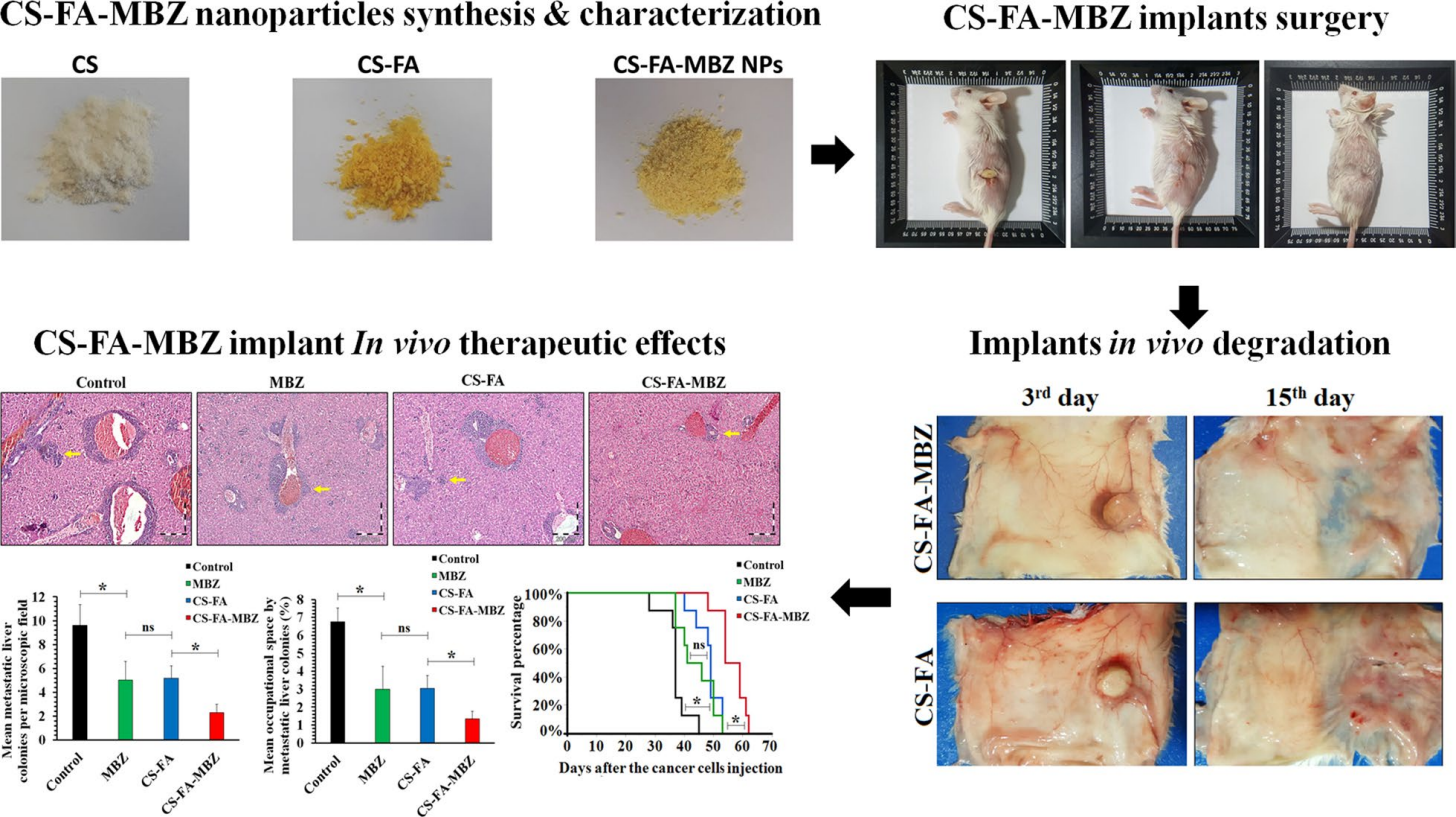

**Supplementary Information:**

The online version contains supplementary material available at 10.1186/s12951-022-01380-2.

## Background

Breast cancer is the most common malignancy among women and the leading cause of cancer-related deaths in the female gender [1]. About 20% of all diagnosed breast cancers are categorized as triple-negative breast cancer (TNBC). This acronym simply means that the tumor doesn’t express estrogen, progesterone, and human epidermal growth factor-2 receptors on its cells’ surface [2]. TNBC is significantly more aggressive and invasive than other subtypes of breast cancer and exhibits higher relapse rates and shorter recurrence period [3]. Therefore, due to the absence of well-defined molecular targets, currently, the only therapeutic approach for TNBC patients is chemotherapy [4, 5]. However, the outcome of chemotherapy with currently approved drugs isn’t satisfying, and poor therapeutic response, severe side effects, and development of multidrug resistance are still big challenges in TNBC chemotherapy [6, 7].

Mebendazole (MBZ) is a well-known antihelminthic drug, with high biocompatibility and low price which has been repurposed for anti-neoplastic treatment [8]. Many studies have reported anti-proliferative, pro-apoptotic, and anti-metastatic effects of MBZ on different cancer cell lines including chemoresistant cancer cell lines [9, 10]. MBZ treatment causes a decrease or complete arrest of tumor growth, significant inhibition of tumor metastasis, and an increase of tumor-bearing mice survival time in different animal models of cancer [11–16]. Anti-neoplastic activities of MBZ can be attributed to inhibition of tubulin polymerization, decrease of tumor angiogenesis, blocking of pro-survival pathways, inhibition of matrix metalloproteinases function, and multi-drug resistance protein transporters activity [8, 17–19]. However, MBZ has limited bioavailability after oral administration as only 20% of the dosage reaches the systemic circulation. This can be related to the low solubility of MBZ, limited absorption efficacy, and extensive first-pass effect following oral administration [20, 21]. Therefore, a high dose of MBZ is required for achieving the therapeutic serum level which can cause severe adverse effects and patients’ non-compliance [22].

Drug-releasing implants have gained lots of attention for controlled drug release in long-term treatments, especially cancer chemotherapy [23, 24]. Implants that contain chemotherapy drugs have exhibited many advantages over intravenous or oral drug administration routes including the elimination of daily multiple injections and maintenance of the steady-state concentration of the drug [25–29]. Biodegradable polymers are the most utilized materials for developing these implants [30]. Chitosan (CS) is a well-known biodegradable biopolyaminosaccharide with a natural origin. The chitosan-based drug carriers and implants exhibit high biodegradability, low toxicity, and appropriate biocompatibility. In addition, chitosan per se has considerable anti-proliferative, pro-apoptotic, anti-angiogenetic, and anti-metastatic effects on different types of tumors [31–33]. Different forms of chitosan-based drug delivery systems have been used and chitosan nanoparticles are one of the most advanced ones for the enhancement of anti-cancer drugs efficacy. They can be defined as spherical, biocompatible, and biodegradable nanostructures with high drug-loading efficacy [34–36]. Chitosan nanoparticles can be used for the controlled release of different types of drugs and enhancement of tumor drug delivery efficacy [37–41]. In addition, chitosan nanoparticles decorating with targeting ligands that can bind to the malignant cells’ surface receptors have received lots of attention for enhancement of tumor drug delivery efficacy [42, 43]. One of the most efficient targeting agents for cancer cells specific targeting is folic acid (FA) due to overexpression of the folate receptor on their membrane in comparison with normal cells. Furthermore, FA has the potential to conjugate directly to polymeric nanoparticles (e.g., through click chemistry), leading to fabrication of novel targeted drug carriers for an efficient delivery [44]. FA has many advantages over other targeting agents, including low molecular weight, simple chemical properties, receptor-medicated endocytosis of the folic acid-decorated carriers, extremely low immunogenicity, overexpression on cancer cells membrane, and limited expression on normal cells’ surface [45–48]. In this regard, many different studies have used FA to decorate their nanostructures for cancer cells targeting and tumor-specific drug release in different cancer models [47, 49]. It should be mentioned that although TNBC cells are known due to the lack of different molecular targets on their membrane, FA is a highly overexpressed receptor on their surface [50–52]. Therefore, FA-modified drug delivery systems can increase the drug concentration at the TNBC tumors site and decline its side effects at normal tissues [53].

In the present study, MBZ-loaded/folic acid-targeted chitosan nanoparticles (CS-FA-MBZ) were used to form cylindrical implants for 4T1 triple-negative breast tumor treatment in BALB/c mice. The therapeutic efficacy of the CS-FA-MBZ implants was investigated after subcutaneous implantation in comparison with Control, MBZ, and CS-FA implants according to 4T1 tumors’ growth progression, metastasis, and tumor-bearing mice survival time. Also, the CS-FA-MBZ implant’s biocompatibility was evaluated by blood biochemical analyzes and vital organs histopathological investigations. In this study, 4T1 murine triple-negative mammary carcinoma was used as an experimental animal model with high similarity to human TNBC. This cell line is a highly tumorigenic and invasive cancer cell line that can spontaneously metastasize from the primary tumor in the mammary gland to multiple distant sites. Also, its metastasis pattern is very similar to that of human breast cancer [54–57]. Although CS-FA nanoparticles have been studied previously as a drug delivery carrier, according to the best of our knowledge, this is the first study to load MBZ in the CS-FA to form CS-FA-MBZ for cancer treatment. In addition, we put on step beyond and designed completely biodegradable scaffolds using the CS-FA-MBZ nanoparticles which by their degradation in the host subcutaneous space, their ingredients (FA-CS-MBZ nanoparticles) will reach the blood circulation for causing their anti-cancer therapeutic effects without any remaining residues at the site of implantation. It should be mentioned that most of the previously studied drug-releasing implants have been implanted at the tumor site or its resected location to prevent its local recurrence [58–60]. However, considerable percentage of cancer patients (including breast cancer patients) experience further metastasis in the distant organs and it is apparent that local release of drug in the tumor site from these scaffolds is not effective for them at all. In addition, some cancer patients are suffering from diffuse metastatic colonies or even unresectable tumor which applying the previously reported drug-releasing scaffolds in the inner of these lesions is practically impossible.

## Materials and methods

### Material

Medium-molecular weight chitosan (190–310 kDa, MMW), Sodium tripolyphosphate (TPP), 1-ethyl-3-(3-dimethylaminopropyl) carbodiimide (EDC), N-hydroxysuccinimide (NHS), folic acid (97%, FA), Tween-80 (a non-ionic surfactant), mebendazole (MBZ), ammonia (25%, NH_4_OH), acetic acid, dimethyl sulfoxide (DMSO), phosphate-buffered saline powder (pH = 7.4 PBS), methanol and acetone were purchased from Sigma-Aldrich (Germany) and used without further purification unless stated otherwise. RPMI 1640, fetal bovine serum (FBS), MTT (3-(4 5-dimethylthiazol-2-yl)-2 5-diphenyltetrazolium bromide), phosphate buffer saline (PBS), penicillin/streptomycin, ethanol (96%, v/v), trypsin–EDTA (0.25%) were purchased from Merck (Germany).

### Conjugation of folic acid to chitosan (CS-FA preparation)

CS-FA was fabricated as reported in the literature [61]. Briefly, 0.5 g of FA and 0.2 g of EDC were initially dissolved in anhydrous DMSO (20 mL) under constant stirring at room temperature (2 h). Then, the solution was dropped into the CS solution 0.5% (w/v) prepared in acetate buffer (0.1 M, pH 4.7) and at room temperature in dark for 16 h. Thereafter, the pH of the solution was adjusted to 9.0 by the addition of NaOH (1.0 M). The resulting precipitate was collected by centrifugation, then purified by dialysis against phosphate-buffered saline (PBS, pH 7.4) for 2 days and against water for another 4 days. Finally, yellow-colored CS-FA products were collected and freeze-dried.

### Preparation of the CS-FA-MBZ nanoparticles

CS-FA-MBZ nanoparticles were synthesized according to the previously reported method with some modifications [62]. Initially, CS-FA (0.1 g) was dissolved in a solution containing acetic acid (1% v/v, 20 mL), then left under stirring at room temperature in dark for 16 h to prepare a solution of CS-FA (0.5% w/v). The pH of the solution was adjusted to 4.8 by the addition of NaOH (1.0 M). Afterward, 250 µL of Tween-80 was added dropwise and left for 2 h under stirring at 45 °C. In the next step, 0.01 g MBZ was dissolved in 0.5 M methanolic hydrochloride [63] and then added to the former solution and stirred for 30 min. Finally, 10 mL TPP aqueous solution (0.5% w/v) was dropped to the CS-FA solution slowly under magnetic stirring (800 rpm) at room temperature for 1 h then the nanoparticles were collected by centrifugation (12,000 rpm, 30 min). The resulting CS-FA-MBZ nanoparticles were lyophilized and stored.

### Calculation of MBZ loading

MBZ was loaded during the formation of CS-FA nanoparticles as reported in the literature [62]. To calculate MBZ encapsulation efficacy (EE%) and loading capacity (LC%), unloaded MBZ content in the supernatant of the last step was determined through a calibration curve of MBZ standard solution by UV–Visible spectroscopy at 234 nm [63]. The MBZ loading ratio of the nanoparticles was calculated by the following Eqs. (1 and 2):

1 $$\mathrm{MBZ \, encapsulation \, efficacy }\left(\mathrm{EE\%}\right)=\frac{\mathrm{ Mass \, of \, the \,loaded \,MBZ }}{\mathrm{Mass \,of \,the \,initial \,MBZ}} \times 100$$

2 $$\mathrm{MBZ \,loading \,capacity }\left(\mathrm{LC\%}\right)=\frac{\mathrm{ Mass \,of \,the \,loaded \,MBZ }}{\mathrm{Mass \,of \,the \,final \,product}} \times 100$$

### In vitro drug release pattern of the CS-FA-MBZ nanoparticles

To evaluate the release behavior of MBZ from CS-FA-MBZ nanoparticles, 5 mg of the CS-FA-MBZ nanoparticles were immersed in PBS solution containing Tween-80 (0.1% w/v) at pH values of 2.2, 5.5, 6.8, and 7.4 at 37 °C in dark under shaking at 100 rpm. The released MBZ was assessed at 0.5 h, 1 h, 2 h, 4 h, 6 h, 8 h, 1 day, 2 days, 3 days, 5 days, and 7 days time points after immersing the nanoparticles in PBS [64]. At each predetermined time point, the nanoparticles were centrifuged (10,000 rpm for 15 min) and the released medium was collected and replaced with equivalent fresh PBS solution. The cumulative percentage of released MBZ was determined by UV–Visible spectrophotometry at 234 nm.

### Nanoparticle’s characterization and implants fabrication

To assess the structure and interaction of CS, CS-FA, and CS-FA-MBZ, Fourier Transform Infrared Spectroscopy (FTIR) was used by a Bruker Equinox 55 spectrometer with the KBr pellets method. To evaluate the size and morphology of nanoparticles, scanning electron microscopy (SEM; FEI ESEM QUANTA 200, MIRAII and MIRAIII Tescan) and transmission electron microscopy (TEM; Philips RM-208, operating voltage: 100 kV) images were acquired. The average size and size distribution of particles were determined by measuring the diameter of 100 particles of SEM images using ImageJ software. The ultraviolet–visible (UV–vis) spectra were recorded by a PerkinElmer Lambda 950 spectrophotometer (wavelength range: 200–800 nm). ^1^H-NMR experiment was recorded on Avance III ultrasheild spectrometer manufactured by Bruker at a field strength of 11.7 T (500 MHz) and the corresponding data was collected using MestReNova software. The zeta potential, hydrodynamic size distribution, and polydispersity of the prepared nanoparticles were measured by Dynamic Light Scattering (DLS) (Malvern Instruments). A well-dispersed aqueous suspension of the prepared nanoparticles was applied and Zeta measurements was performed at pH value of 7.5. Each experiment was carried out in triplicate and data were presented as means ± standard deviations. For fabricating an implant, the adequate mass of the synthesized CS-FA-MBZ nanoparticles (according to the mouse body weight and its needed dosage of MBZ) were pressed in a steel die at 1500 psi to form cylindric implants (usually 6 mm diameters and 3 mm height).

### Cell viability assay

MTT assay was employed for the cell viability evaluation according to our previous studies [56, 65]. The 4T1 and L929 cells were separately seeded into 96-well culture plates at 5 × 10^3^ cells/well density. After 24 h incubation, different concentrations (0, 0.5, 1, and 1.5 µM) of MBZ, CS-FA, and CS-FA-MBZ were dissolved in culture medium and added to the wells. The cells were incubated for 48 h and then, the culture media was replaced with RPMI culture medium containing 0.005% MTT solution. After 4 h incubation in the standard cell culture incubator, the medium was discarded and the precipitated formazan crystals were dissolved in dimethyl sulfoxide (DMSO). At last, an absorbance Microplate Reader (BioTek-ELX800, USA) was used to measure the absorbance of the wells at 570 nm wavelength. Subsequently, the below-mentioned Eq. (3) was used to calculate the cell viability percentage of the treated wells in comparison with the control wells (0 µM). The experiment was repeated three times and at least six wells were used for each concentration.

3 $$Cell\,viablity \left(\%\right)=\frac{\left(OD\,Sample-OD\,Blank\right)}{(OD\,Control-OD\,Blank)}\times 100.$$

### Animal ethics, care, and handling

All animal experiments complied with the ARRIVE guidelines and were conducted according to the guidelines of the European Communities Council Directive (2010/63/UE) and the Isfahan University of Medical Sciences for the care and use of laboratory animals. Likewise, all the procedures, protocols, and steps were approved by the ethics committee of the Isfahan University of Medical Sciences (IR.MUI.RESEARCH.REC.1399.125). Female BALB**/**c mice (weight: 25 ± 2 g) were purchased from the Pasteur Institute of Tehran, Iran. The mice were acclimatized to the laboratory environment (24 ± 2 °C temperature, 50 ± 10% relative humidity, and 12 h light/12 h dark cycles) for 14 days before involving in the experiments. All mice were fed sterilized standard mouse chow and water ad libitum. Overdose of Ketamine-Xylazine (KX) solution through intraperitoneal injection was used for the mice sacrifice.

### Tumor implantation

4T1 cancer cells (murine mammary carcinoma) were purchased from the Pastor Institute of Tehran, Iran. The cells were cultured in RPMI 1640 medium containing 10% fetal bovine serum (FBS). The cells were incubated at 37 °C in a humidified incubator in 5% CO_2_ atmosphere. When the cells reached adequate numbers, they were harvested from culture flasks by trypsin and washed three times with PBS. The mice were injected with 2 × 10^6^ cells suspended in 50 µL of FBS-free DMEM-F12, subcutaneously (s.c.) into the left 4th abdominal mammary fat pad.

### Tumor-bearing mice grouping and therapeutic approaches

For this experiment 32 tumor-bearing mice were used. When the tumors’ volume reached 50–70 mm^3^ (3rd day after the cancer cells injection), mice were divided into four groups (n = 8) including (1) Control, (2) MBZ (40 mg/kg, oral administration, twice a week for 2 weeks), (3) CS-FA implants, (4) CS-FA-MBZ implants. The tumor-bearing mice in the 2nd group were treated with oral administration )p.o.( of MBZ (40 mg/kg, twice a week for 2 weeks according to previous studies [66]) from the 3rd day after the cancer cells injection. In the 3rd and 4th groups, the tumor-bearing mice were anesthetized with intraperitoneally injection of Ketamine-Xylazine (KX) solution (Ketamine: 100 mg/kg, Xylazine: 10 mg/kg). The left flank was shaved and scrubbed with betadine. The scrub solution was wiped away from the surgical site with alcohol 70% and covered with a sterile drape. Then, a small (~ 1 cm) incision was made and the implant was embedded under sterile conditions, and the skin was stitched with nylon (4–0). All the operations were done under complete anesthesia. To manage post-surgical pain, ketoprofen (5 mg/kg) was administered subcutaneously until the next 72 h. It should be mentioned that mice in the Control and MBZ group underwent the same surgery at the same day as two other groups and post-operative pain management protocol to prepare the same condition in all groups. The mice were monitored daily for prolonged signs of pain, weight loss, or surgical site infections. If any signs of pain, wounds infection, massive necrosis, and hemorrhage, diffuse metastasis were observed during any steps of the study, the mice were sacrificed by KX overdose. In the Control group, one incision was made at the left flank of the tumor-bearing mice and sutured without implantation of any implants. To determine tumors’ growth progression, the greatest longitudinal diameter (length) and the greatest transverse diameter (width) of the tumors were measured every 3 days until the 18th after cancer cells injection. Then, the tumor’s volume was calculated by the tumor volume Eq. (4). For survival analysis, the tumor-bearing mice were observed for 70 days after treatment administration. The animals’ death was recorded every day. It should be mentioned that standardized humane endpoints based on the current guidelines for endpoints in animal tumor studies were used [67–69].

4 $$Tumor\,volume=\frac{\left(Tumor\,length\right)\times {(Tumor\,width)}^{2}}{2}$$

### 4T1 breast tumors’ metastasis

For this experiment, 20 tumor-bearing mice were involved (n = 5) and the groups and therapeutic methods were completely the same as the previous section. The mice were sacrificed by overdose of ketamine/xylazine 30 days after cancer cell implantation and their livers were harvested and fixed in 10% neutral buffered formalin solution. An automatic tissue processor (Sakura, Japan) was employed to process the fixed samples. Then, a microtome (Leica Biosystems, Germany) was utilized to cut 4 µm thickness serial sections from the paraffin-embedded blocks. The sections were stained with Hematoxylin & Eosin (H&E) staining protocol according to previous studies [57, 70]. A minimum of 10 random microscopic fields was observed under the 10 × objective lens of a light microscope (Olympus, Japan) to report the mean number of metastatic colonies per microscopic field of the liver. Furthermore, the occupied area by metastatic colonies in each microscopic field of the liver (magnification × 100) was quantified by the Qupath software. The mean percentage of occupied space by liver metastatic colonies in each microscopic field was reported for each sample.

### Histopathology and blood biochemical assays

For evaluating the safety of the subcutaneous CS-FA-MBZ implants, 20 healthy mice were involved and randomly divided into four groups (n = 5) including (1) Control, (2) MBZ, (3) CS-FA, and (4) CS-FA-MBZ implants according to the “Tumor-bearing mice grouping and therapeutic approaches” section. The mice were monitored for general appearance and behavioral parameters for 30 days. They were under close monitored for any signs of toxicity and behavioral changes including weakness, salivation, anorexia, diarrhea, aggressiveness, eyes and ears discharge, noisy breathing, activity, convulsion, cachexia, pain, or any signs of illness in each group for 30 days [71]. On the 30th day, the mice were sacrificed and blood urea nitrogen (BUN), creatinine (Cr), alanine aminotransferase (ALT), and aspartate aminotransferase (AST) levels were measured in the discarded serums [72]. In addition, lungs, kidneys, liver, and spleen were harvested and fixed, processed, and H&E stained. Histological photographs were obtained using a digital light microscope (Olympus, Japan).

### Statistical analysis

The statistical analyzes were performed using one-way analysis of variance (ANOVA) with Tukey’s post-hoc test by JMP 14.0 software (SAS Institute, Japan). The results were statistically significant at *P* < 0.05 (*: *P* <  0.05, ns: not significant). All values were expressed as the mean ± standard deviation.

## Results and discussion

### Fabrication and characterization of the CS-FA-MBZ nanoparticles

**Fig. 1:**
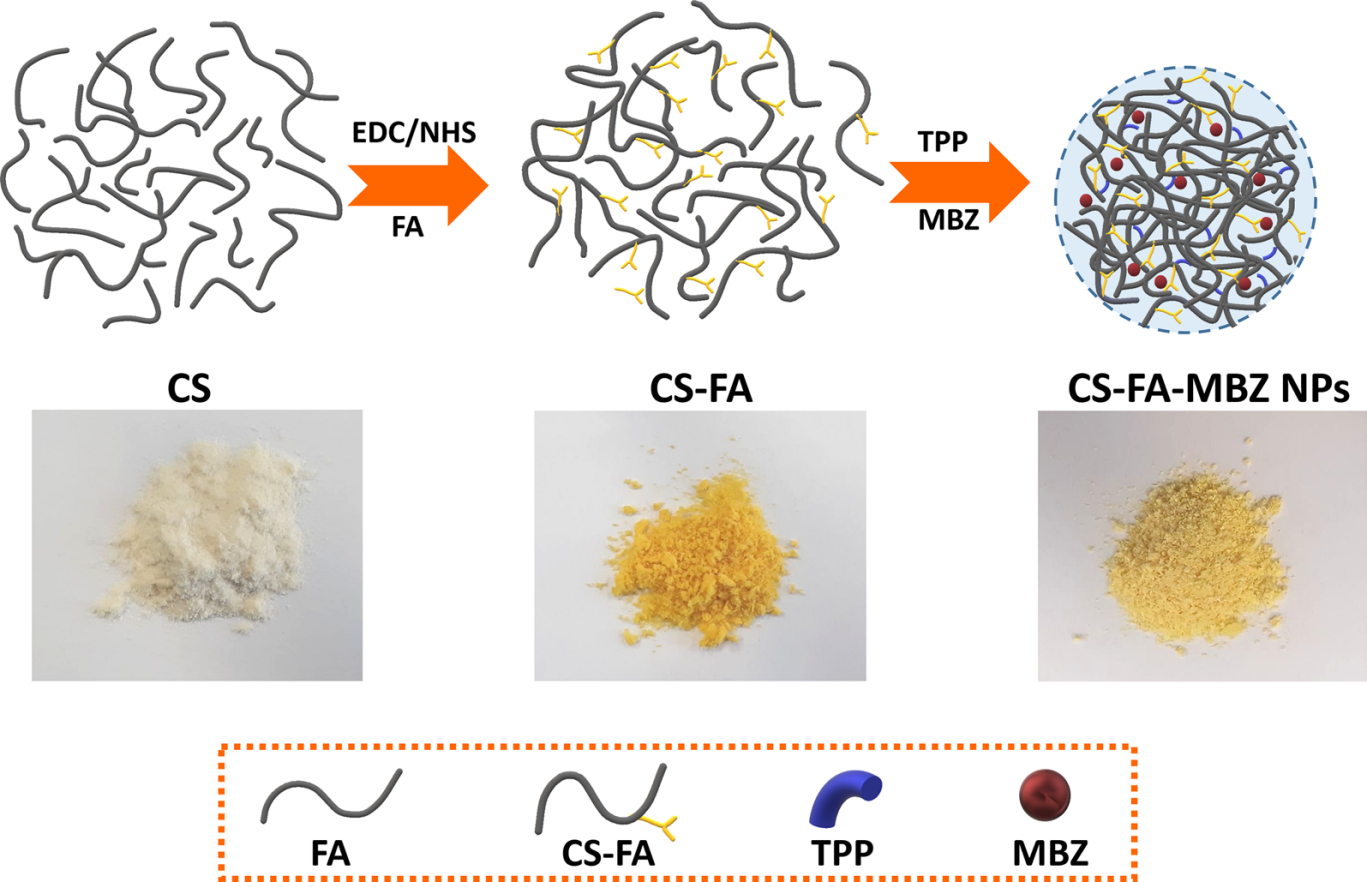
Sequential steps for preparation of the CS-FA-MBZ nanoparticles

To fabricate CS-FA-MBZ nanoparticles, at first, folic acid was conjugated to chitosan in the presence of EDC as carboxyl activating agent to produce CS-FA (Additional file 1: Figure S1). The activated carboxyl moiety of FA was covalently linked to the amine groups of CS [73]. At the next step, the cross-linking reaction between CS-FA and TPP led to the formation of nanoparticles as the negatively charged TPP was electrostatically adsorbed to the positively charged free protonated amine groups of the CS-FA. Subsequently, MBZ was encapsulated during the synthesis process (Fig. 1).

**Fig. 2:**
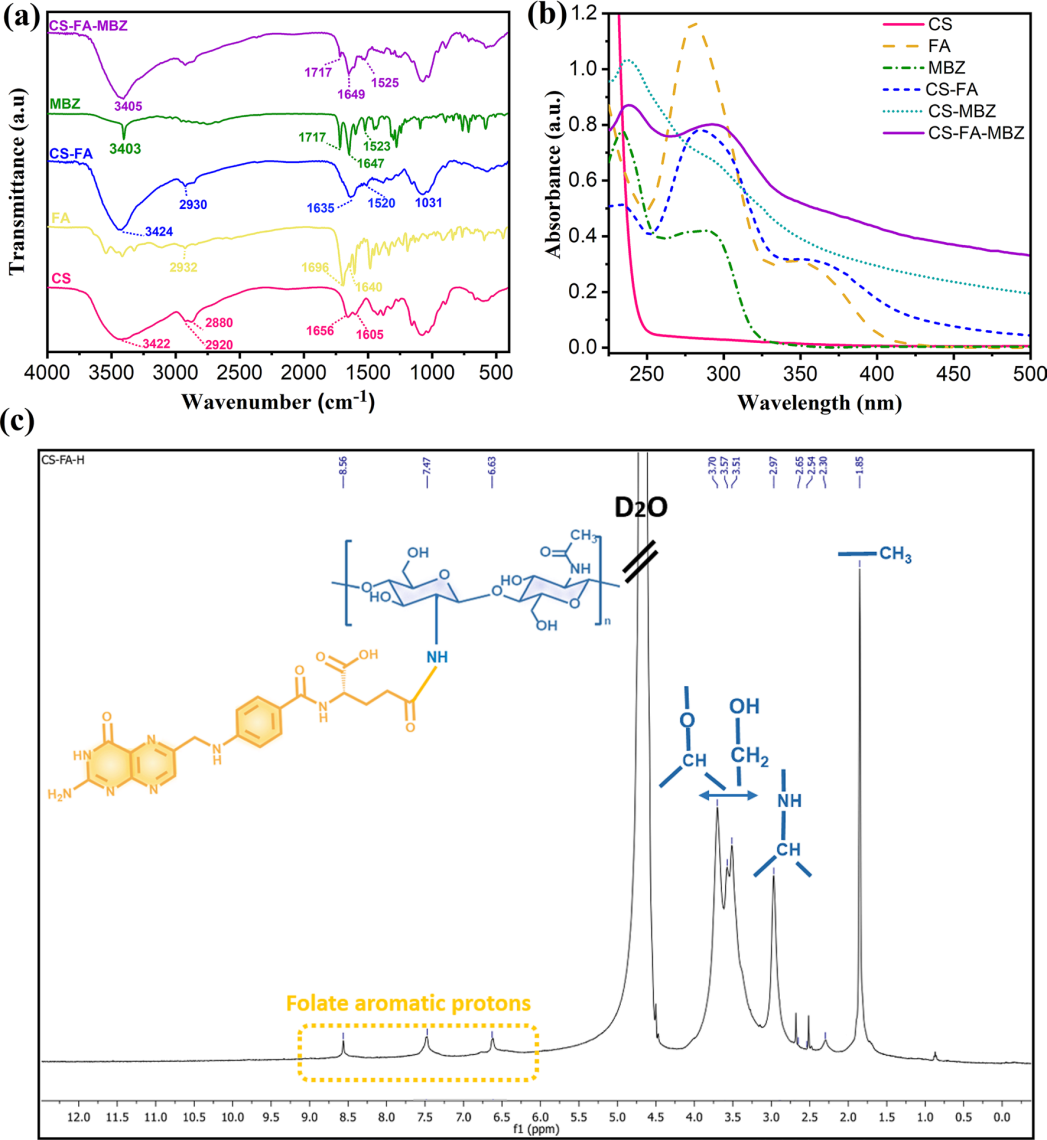
**a** FTIR spectrum of CS, FA, CS-FA, MBZ, and CS-FA-MBZ nanoparticles. **b** UV–Vis spectra of CS, FA, MBZ, CS-FA, CS-MBZ, and CS-FA-MBZ. **c** ^1^H-NMR spectrum of CS-FA in deuterated acetic acid (CD_3_COOD) in D_2_O

The successful introduction of folate to CS chains was initially evaluated by FTIR (Fig. 2a). As shown in Fig. 2a, the characteristic bands of CS located at 3422 cm^−1^ assigned to O–H stretching vibration overlapped with N–H stretching mode. The bands at 2920 and 2880 cm^−1^ could be attributed to the C-H stretching vibrations of CS. Moreover, the peaks that appeared at 1656 and 1605 cm^−1^ correspond to the C–O stretching vibration of amide I and N–H bending vibration of amide II, respectively [74]. FTIR spectrum of free FA showed characteristic bands located at 1696 and 1640 cm^−1^ related to the C=O stretching vibration of the carboxyl group and N–H bending vibration of CONH, respectively [75]. In the CS-FA spectrum, the absorption peaks at 1635 and 1031 cm^−1^ could be attributed to the vibration of C–N [74]. The amid band at 1656 cm^−1^ of CS shifted to 1635 cm^−1^ because of overlapping with the newly formed amide bond, and also a new N–H bending vibration located at 1520 cm^−1^ confirmed the successful conjugation of FA to CS [76]. Moreover, UV–Vis spectra of CS, FA, and CS-FA are shown in Fig. 2b. CS had no absorption while the characteristic absorption bands of FA were appeared at 280 and 350 nm which could be related to the typical π → π* transition of its pterin ring. CS-FA exhibited the FA absorption bands with a slight shift to longer wavelength (284 and 360 nm) indicating the conjugation of CS to FA. Conjugation process was further demonstrated by ^1^H-NMR analysis (Fig. 2c). Signals appeared at δ 1.85, 2.97 and 3.51–3.70 ppm which could be attributed to the resonance of the –COCH_3_, –CH–NH–, and –CH_2_–O– groups in CS, respectively [77]. Presence of signals at δ 2.54 and 2.65 ppm could be related to methylene groups of FA attached to the new amide bond [61, 76]. Moreover, the resonance of the folate aromatic protons observed at 6.5–8.5 ppm, confirmed the successful conjugation of CS to FA. CS-FA was dissolved in deuterated acetic acid (CD_3_COOD) in D_2_O. Therefore, the acetic peak was apparent at δ 2.30 ppm [78]. Taken together, FTIR, UV–Vis, and ^1^H-NMR analyses verified the successful conjugation process and their results were consistent with the previous studies [79].

Thereafter, MBZ, as a hydrophobic drug, was loaded into the CS-FA nanoparticles (CS-FA-MBZ). FTIR and UV–Vis analyses were applied for verification of MBZ loading as well. FTIR results (Fig. 2a) showed the N–H stretching and bending vibrations of MBZ at 3403 and 1523 cm^−1^ which were observed for CS-FA-MBZ at 3405 and 1525 cm^−1^, respectively. Furthermore, the stretching vibration of amide I of the carbamate group of MBZ (1717 cm^−1^) appeared at the CS-FA-MBZ nanoparticles spectrum in the same location, indicating successful MBZ loading [80]. The UV–Vis spectrum of MBZ (Fig. 2b) showed the characteristic absorption band at 234 nm which was observed for CS-MBZ as well. This band along with the absorption band of FA at ~ 280 nm with a slight shift to longer wavelengths was detected in the spectrum of CS-FA-MBZ, indicating the appropriate MBZ loading into the CS-FA nanoparticles.

**Fig. 3:**
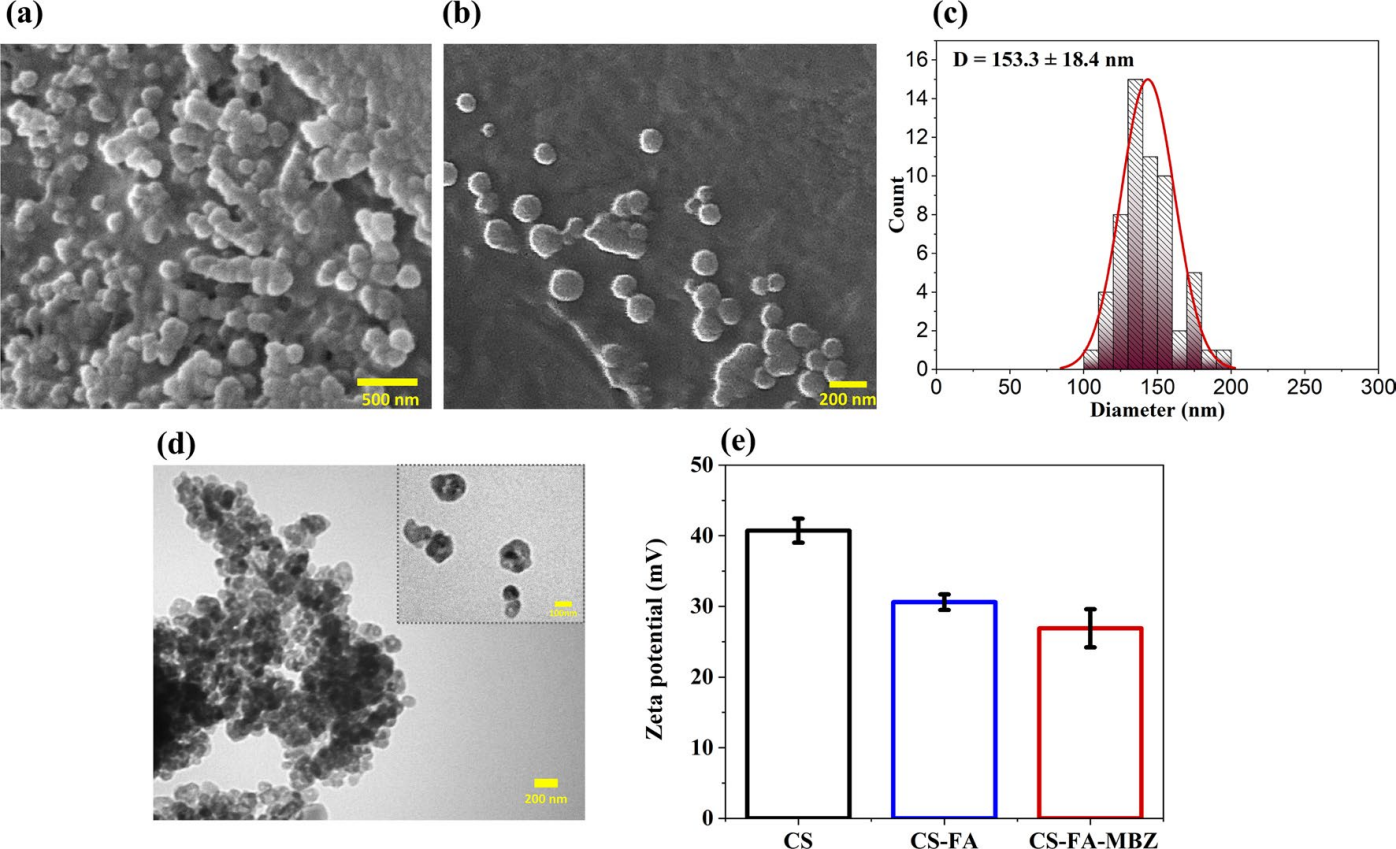
**a**, **b** SEM images of the CS-FA-MBZ nanoparticles with different magnifications. **c** Size distribution of the CS-FA-MBZ nanoparticles determined by ImageJ software considering 100 particles in corresponding SEM images. **d** TEM images of CS-FA-MBZ nanoparticles. **e** Zeta potential distribution histogram of CS, CS-FA, and the CS-FA-MBZ nanoparticles

Figure 3a and b show SEM images of the CS-FA-MBZ at different magnifications, in which the nanoparticles were uniform, with mainly spherical morphology. Figure 3c shows the corresponding particle size distribution histograms (dry state) obtained by measuring the size of 100 nanoparticles in the SEM images by the “Image J” software. A narrow size distribution with a mean size of 153.3 ± 18.4 nm was measured. Moreover, the particle size distribution of the CS-FA-MBZ was determined by DLS measurement (wet state). The hydrodynamic diameter of the nanoparticles was measured 182 ± 12.1 nm (Additional file 1: Figure S2) with a low polydispersity index (PDI < 0.2) illustrating a narrow size distribution which was consistent with the results obtained from the SEM. In addition, TEM images of CS-FA-MBZ nanoparticles are shown in Fig. 3d. The zeta potential distribution histograms of CS, CS-FA and CS-FA-MBZ at pH value of 7.5, exhibit that the positive charge of chitosan due to the presence of the amine groups decreases with the conjugation of FA (Fig. 3e). It can be attributed to the interaction of FA molecules with these amine groups of CS leading to the neutralization of the potential value [81]. Additionally, the results depict that the encapsulation of MBZ into the CS-FA nanoparticles induces a noticeable change of zeta value of the final nanoparticles with the preserved positive value of + 27 mV.

### MBZ loading

After successful loading of MBZ into CS-FA nanparticles (Fig. 2a, b), the encapsulation efficacy (EE) and loading capacity (LC) of MBZ were calculated by plotting the standard calibration curve with a linear curve fit equation (Additional file 1: Figure S3). The value of EE and LC were 57.7% and 10.5%, respectively. This fact demonstrates the valuable capability of the target system for MBZ loading.

### In vitro MBZ release pattern

**Fig. 4:**
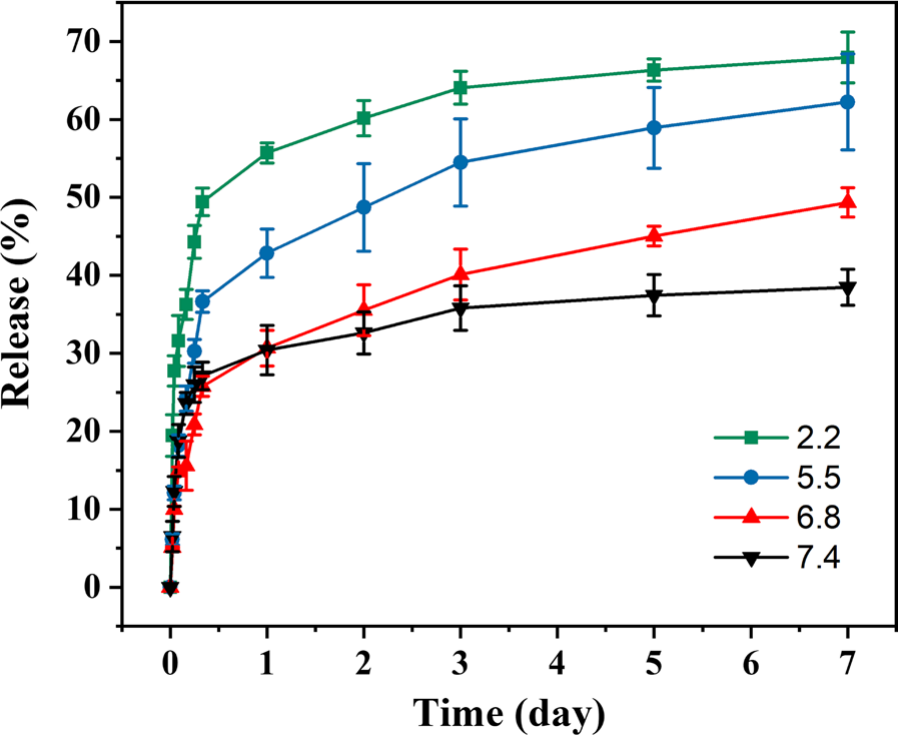
Release profiles of MBZ from CS-FA-MBZ nanoparticles at pH = 2.2, 5.5, 6.8, and 7.4. Data are expressed as mean ± SD (n = 3)

The release pattern of MBZ from CS-FA-MBZ was evaluated at different pH values including, 2.2, 5.5, 6.8, and 7.4 to mimic various microenvironments. The MBZ release pattern was monitored at different time points including, 0.5 h, 1 h, 2 h, 4 h, 6 h, 8 h, 1 day, 2 days, 3 days, 5 days, and 7 days after immersing the CS-FA-MBZ nanoparticles in PBS according to previous studies [64]. As Fig. 4 illustrates, the CS-FA-MBZ nanoparticles exhibited a continuous and sustained MBZ release profile at different pH values started with a burst release within 6 h followed by a gradual release within the next 7 days. The initial fast release could be related to the MBZ loaded near the surface of nanoparticles [75]. Besides, the CS-FA-MBZ nanoparticles revealed a pH-responsive behavior for MBZ release. At the 7th day, the release of MBZ was ~ 68%, 62%, 49%, and 38% at pH values of 2.2, 5.5, 6.8, and 7.4, respectively. This behavior could be assigned to the high swelling ability of CS which was induced by protonation of the amine groups of the polymer in the acidic environment [75]. Considering the acidic microenvironment of cancerous tissues, the pH-sensitive behavior of the CS-FA-MBZ nanoparticles can improve their capability for tumor-specific drug release. The pH-sensitive property of the CS-FA-MBZ nanoparticles can accelerate MBZ release in the acidic microenvironment of tumor while avoids its premature release in the normal tissues’ microenvironment which can reduce the probable adverse effects to the normal tissues[82].

### Cytotoxic effect of CS-FA-MBZ nanoparticles on the breast cancer and normal cells

**Fig. 5:**
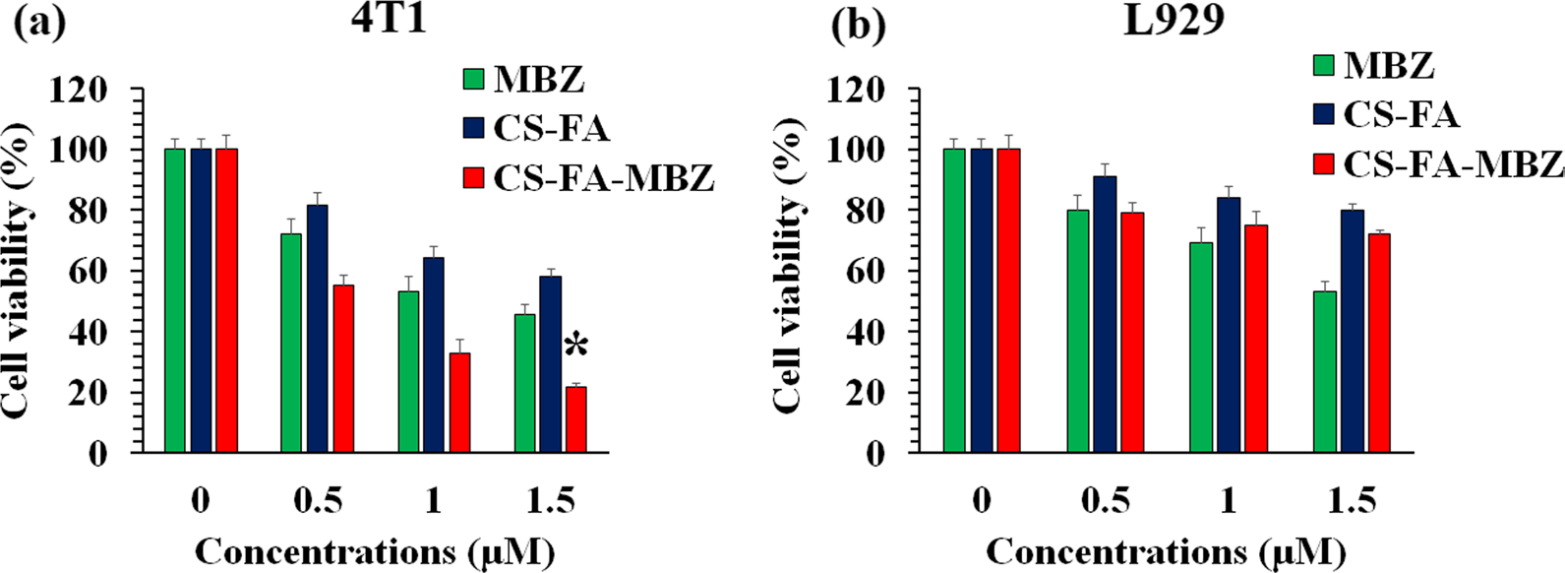
Cytotoxic effect of different concentrations (0, 0.5, 1, and 1.5 µM) of MBZ, CS-FA, and CS-FA-MBZ nanoparticles on **a** 4T1 breast cancer and **b** L929 normal cells viability after 48 h incubation according to MTT assay

The 4T1 murine triple-negative mammary cancer and L929 normal murine fibroblast cell lines were treated with different concentrations (0, 0.5, 1, and 1.5 µM) of MBZ, CS-FA, CS-FA-MBZ nanoparticles. As Fig. 5 illustrates, MBZ and CS-FA nanoparticles exhibit anti-cancer properties which is dose dependent. On the other hand, the most significant decrease in the 4T1 cancer cells viability percentage was observed in the highest dose of the CS-FA-MBZ nanoparticles-treated wells which demonstrates the increased anti-cancer effectiveness of MBZ after loading in the CS-FA platform. This can be attributed to the folic acid targeting of the nanoparticles which increases the internalization of loaded MBZ into the cancer cells and also, the anti-cancer effect of CS nanoparticles per se which is consistent with the previous publications [83]. Taking together, CS-FA-MBZ exhibits not only significantly more cytotoxic effects on the 4T1 cancer cells in comparison with the CS-FA nanoparticles or pure MBZ but also, lower toxicity on the L929 normal cells rather than MBZ.

### The CS-FA-MBZ implants effect on the 4T1 breast tumors’ growth progression

**Fig. 6:**
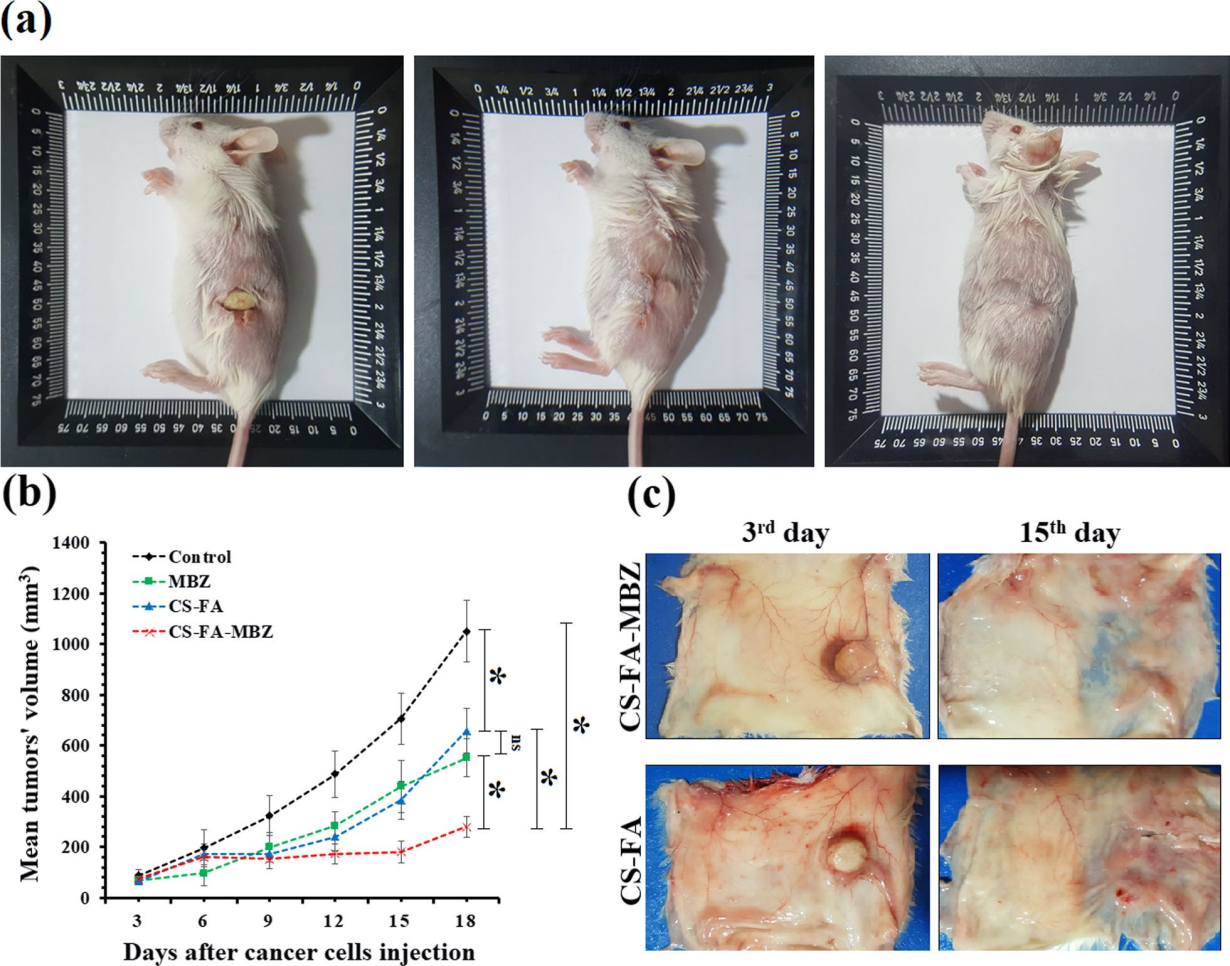
**a** Subcutaneous implantation of the CS-FA-MBZ implants. A small incision was made at the left flank of mice and the implant was placed inside it. The incision was sutured. The implantation site on the 18th day after cancer cells injection (15th day after surgery). **b** Tumors’ growth progression in different groups including Control, MBZ, CS-FA, and CS-FA-MBZ from the 3rd to 18th day after cancer cells injection (not significant: ns, *: *P* < 0.05). **c** Harvested skin of the implants-bearing mice at the 3rd and 15th days after implantation which equates the 6th and 18th days after cancer cells injection, respectively

As Fig. 6a illustrates, the CS-FA and CS-FA-MBZ implants were s.c. implanted in the left flank of the tumor-bearing mice inside a small incision on the 3^rd^ day after the cancer cell injection. The implants were completely palpable even after suturing the incision. The implants completely degraded until the 18th day in both CS-FA and CS-FA-MBZ groups and nothing was palpable at the implantation site. This means that the implants were dissociated and release their composing agents means CS-FA-MBZ nanoparticle.

The therapeutic effect of the CS-FA-MBZ implants on inhibition of 4T1 tumors’ growth progression was evaluated by serial measurement of tumor’s diameters and compared with the Control, MBZ (40 mg/kg, oral administration, twice a week for 2 weeks), and CS-FA groups (Fig. 6b). All treatments were initiated from the 3rd day after cancer cells injection. As Fig. 6b illustrates, the CS-FA-MBZ implants could significantly inhibit the breast tumors growth progression in comparison with all other groups. On the last day of tumors’ volume monitoring (18th day after cancer cells injection), the mean tumors’ volume at the CS-FA-MBZ group (280.6 ± 42.2 mm^3^) was significantly (*P* < 0.05) lower than the Control (1050.5 ± 120.7 mm^3^), MBZ (552.4 ± 76.1 mm^3^), and CS-FA (658.3 ± 88.1 mm^3^) groups. The CS-FA-MBZ implants caused about 73.3%, 49.2%, 57.4% decrease in the mean tumors’ volume in comparison with the Control, MBZ, and CS-FA groups, respectively. Therefore, CS-FA-MBZ implants exhibit high efficacy in inhibiting breast tumors growth.

**Fig. 7:**
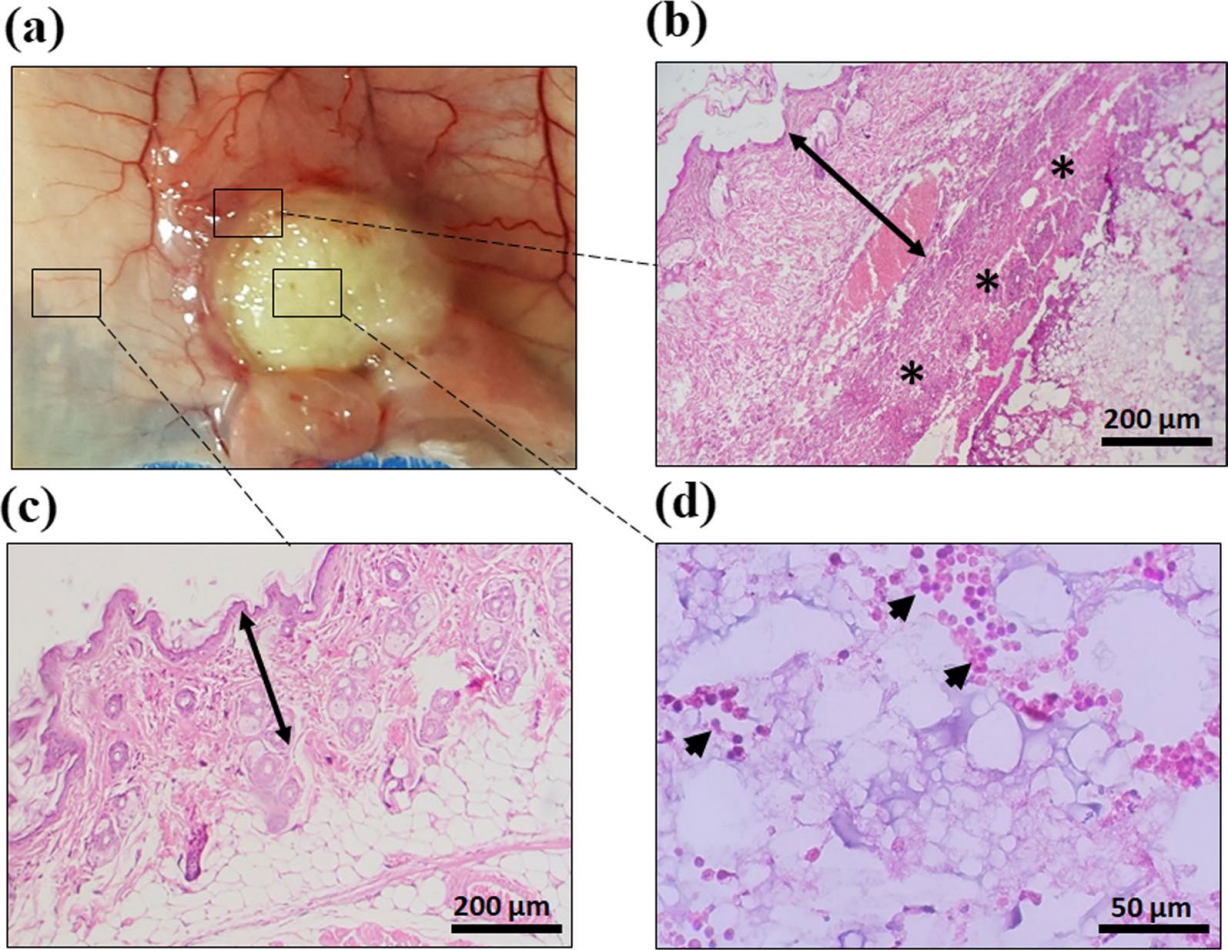
Mouse body response to the subcutaneously implanted CS-FA-MBZ at 3rd day after implantation according to histopathology analysis. **a** A close-up view of a CS-FA-MBZ implant 3rd day after implantation and histopathological evaluation of **b** the implant-surrounding membrane, **c** tissues far away from the implantation site, and **d** tissues at the bed of the implantation site. The two head arrows, asterisk, and one-head arrows indicate derma, the implant’s surrounding membrane, and infiltrating immune cells, respectively

On the 6th and 18th days (3rd and 15th days after surgery, respectively), some implants-bearing mice were sacrificed to observe what is happening on the CS-FA and CS-FA-MBZ implants’ site (Fig. 6c). As illustrated in Fig. 6c, the implants were surrounded by a thin transparent membrane on the 6th day. On the 18th day, the implants were completely degraded and disappeared. As Fig. 7 illustrates, histopathological evaluations of the implantation site on the 6th day demonstrated that the surrounding membrane consisted of connective tissue. Also, limited mononuclear cells infiltration was observed at the implants’ bed (Fig. 7).

### The CS-FA-MBZ implants effect on metastasis and tumor-bearing mice survival time

**Fig. 8:**
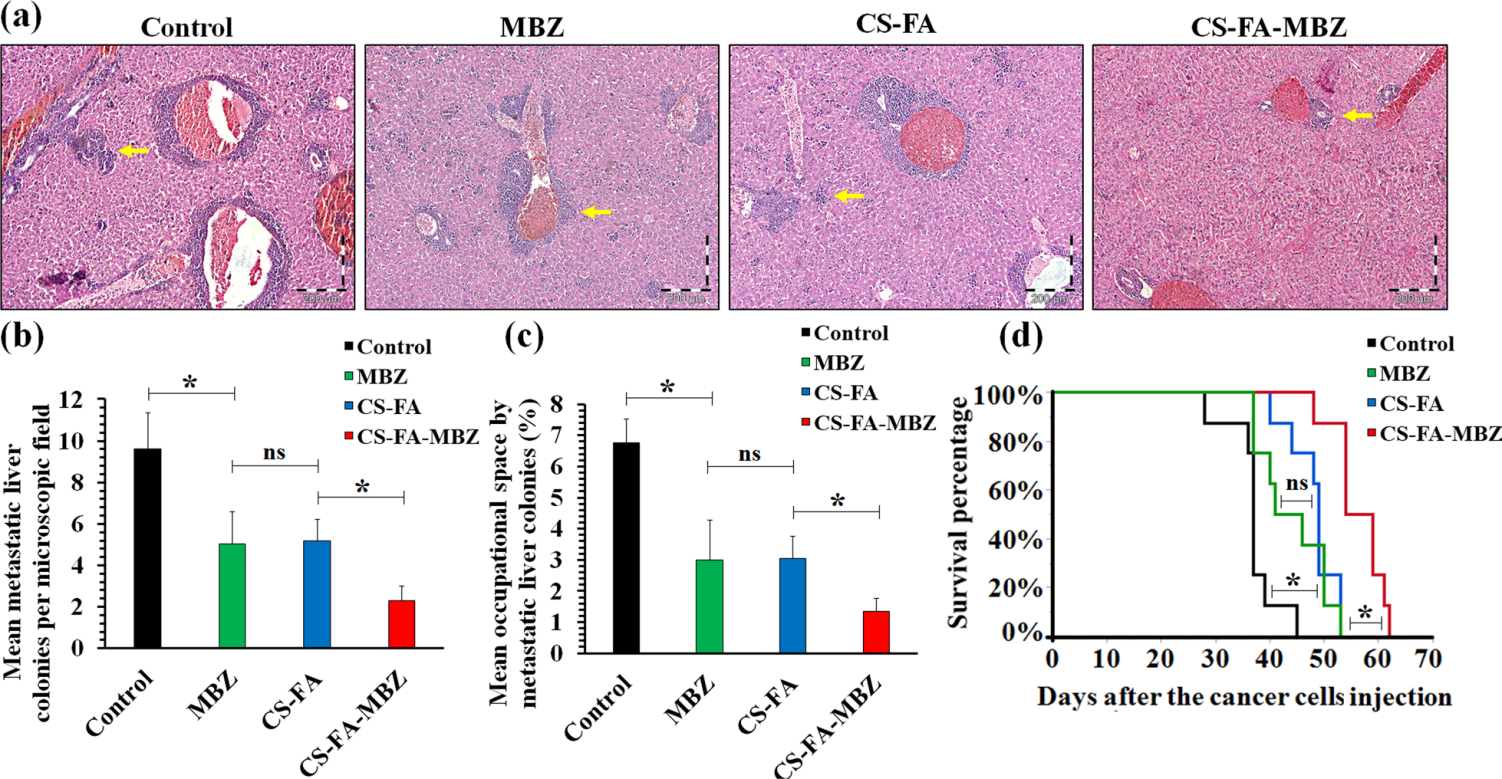
Metastatic burden and survival time of the tumor-bearing mice at different treatment groups. **a** One microscopic field of H&E-stained sections of livers in each group was illustrated as the sample. **b** The average number of metastasis colonies per microscopic field of the tumor-bearing mice’s liver 30 days after the cancer cells injection. The yellow arrows indicate a liver metastatic colony. **c** Mean percentage of the occupied space per microscopic field of the tumor-bearing mice’s liver 30 days after the cancer cells injection. **d** The survival time of tumor-bearing mice in different groups. (Not significant: ns, *: *P* < 0.05)

Metastasis is the main cause of cancer-related deaths. The formation of metastatic colonies at vital organs like the liver disrupts their function and causes organ failure [84]. Therefore, to evaluate the effect of the CS-FA-MBZ implants on metastasis formation, H&E sections were used to count the metastatic colonies at tumor-bearing mice liver after 35 days from cancer cells injection (Fig. 8). Histopathological evaluations demonstrated significant inhibition of the liver metastatic colonies formation at the CS-FA-MBZ (2.3 ± 0.7) treated mice in comparison with the Control (9.6 ± 1.7), MBZ (5.0 ± 1.5), and CS-FA (5.2 ± 1) groups (Fig. 8a and b). Besides, the metastatic colonies occupied significantly (*P* < 0.05) lower space in the liver sections (per microscopic field) of the CS-FA-MBZ treated group in comparison with the other groups (Fig. 8c). In addition, the CS-FA-MBZ group exhibited about 52.1%, 27.3%, and 17% more survival time (days) in comparison with the Control, MBZ, and CS-FA groups, respectively (Fig. 8d). This increase in the tumor-bearing mice survival time can be attributed to significant inhibition of 4T1 tumors’ growth and metastasis.

### Biocompatibility of the subcutaneous CS-FA-MBZ implants

**Fig. 9:**
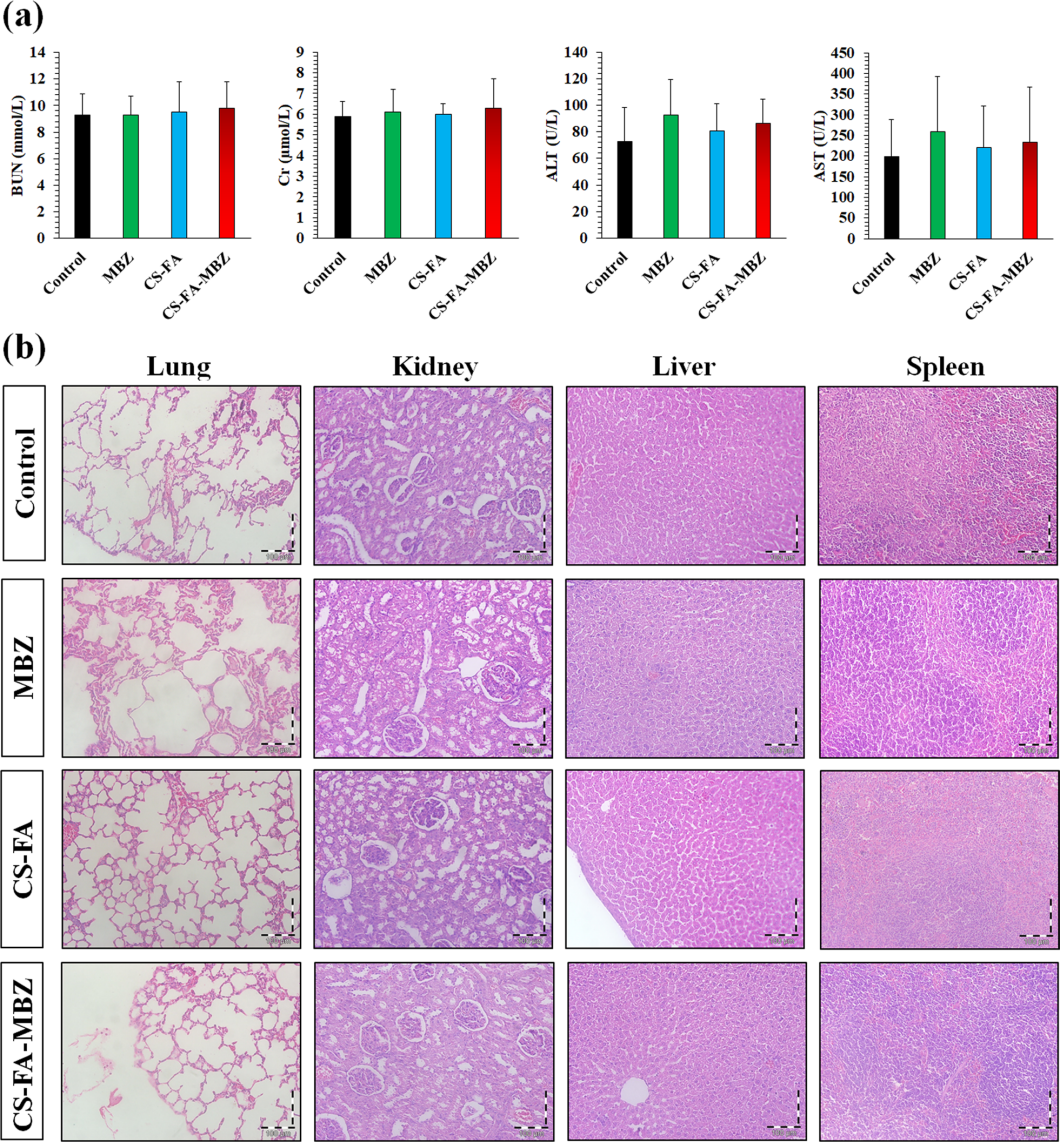
Assessment of the CS-FA-MBZ implants biocompatibility according to blood biochemical analyzes and histopathological evaluation of vital organs. **a** Serum level of BUN, Cr, ALT, and AST of the CS-FA-MBZ implanted mice (n = 5) 30 days after implantation in comparison with the Control, MBZ, and CS-FA groups. There is no statistical difference between the groups. **b** H&E-stained sections of lungs, kidneys, spleen, and liver of the CS-FA-MBZ implanted mice (n = 5) 30 days after implantation in comparison with the Control, MBZ, and CS-FA groups

The safety and biocompatibility of implants are very important for clinical application. Therefore, the CS-FA-MBZ implants’ safety and biocompatibility were evaluated in comparison with the Control, MBZ, CS-FA group in non-tumor-bearing mice. The treatment protocol was the same as “Tumor-bearing mice grouping and therapeutic approaches” section for each group. The mice were exactly monitored according to general appearance and behavioral parameters, blood biochemical analyzes, and histopathological evaluation of vital organs. No sign of change in the mice's appearance, behavioral pattern, and food intake were observed during the 30 days (Additional file 1: Tables S1) in none of the groups. On the 30th day, the animals were sacrificed and their plasma was collected for biochemical (Fig. 9a) analyses and the vital organs were harvested for histopathological exams (Fig. 9b). No significant sign of organ damage was observed in either H&E sections and blood biochemical analyzes. Chitosan nanoparticles are the main component of CS-FA-MBZ implants. Chitosan is a natural biodegradable biopolymer. The enzymatic degradation of chitosan causes its transformation to some components which are completely safe. Many enzymes have the ability to degrade chitosan and the most well-known one is lysozyme as a non-specific protease. This enzyme which presents in all mammalian tissues and fluids, plays a key role in degradation of chitosan-based implants in vivo. It targets the acetylated residues of chitosan polymer and degrades chitosan to non-toxic oligosaccharides which can be excreted or incorporated to glycosaminoglycans and glycoproteins [30, 85–87]. Also, eight human chitinases (in the glycoside hydrolase 18 family) have been identified, three of which have shown enzymatic activity [88]. On the other hand, in the MBZ group which were under treatment with oral administration of MBZ a mild increase in the liver enzymes was observed which is a routine side effect of MBZ [89]. This can be related to the hepatotoxic effect of MBZ which was not detected at the CS-FA-MBZ implants groups. Taking together, CS-FA-MBZ implants system can decrease the side effects of MBZ and cause significant therapeutic efficacy in the TNBC tumor-bearing mice.

## Conclusions

Chemotherapy drugs loaded implants have exhibited higher efficacy in comparison with common routes of drug administration. In the present study, folic acid-targeted chitosan nanoparticles were used as a carrier to increase MBZ therapeutic efficacy in the triple-negative breast cancer-bearing BALB/c model. A definite amount of MBZ-loaded CS-FA nanoparticles were compressed to form cylindrical CS-FA-MBZ implants which were subcutaneously implanted at mice’s flank. The CS-FA-MBZ implants could significantly inhibit 4T1 breast tumor growth and metastasis. Therefore, these biodegradable and biocompatible implants can be an appropriate choice for further experiments in breast cancer and other cancers treatment.

## Supplementary Information

**Additional file 1: Figure S1. **Schematic illustration of folic acid conjugation to chitosan in presence of EDC as carboxyl activating agent to produce CS-FA. **Figure S2.** The CS-FA-MBZ nanoparticles hydrodynamic size distribution according to DLS measurements. **Figure S3.** Standard calibration curve of MBZ. **Table S1.** General appearance and behavioral observations for Control and CS-Fa-MBZ groups (n = 5).

## Data Availability

All datasets are available upon reasonable request.

## Abbreviations

TNBC: Triple-negative breast cancer
MBZ: Mebendazole
FA: Folic acid
CS: Chitosan
KX: Ketamine-xylazine
