# Supplementary material for: Biodegradable and biocompatible subcutaneous implants consisted of pH-sensitive mebendazole-loaded/folic acid-targeted chitosan nanoparticles for murine triple-negative breast cancer treatment
Source: J Nanobiotechnology. 2022 Mar 31;20:169. doi: 10.1186/s12951-022-01380-2 (PMC8973744; doi:10.1186/s12951-022-01380-2)
Supplement: Supplementary file 1 — Additional file 1: Figure S1. Schematic illustration of folic acid conjugation to chitosan in presence of EDC as carboxyl activating agent to produce CS-FA. Figure S2. The CS-FA-MBZ nanoparticles hydrodynamic size distribution according to DLS measurements. Figure S3. Standard calibration curve of MBZ. Table S1. General appearance and behavioral observations for Control and CS-Fa-MBZ groups (n = 5). [file 12951_2022_1380_MOESM1_ESM.docx]

**Biodegradable and Biocompatible Subcutaneous Implants Consisted of pH-sensitive Mebendazole-Loaded/Folic acid-targeted Chitosan Nanoparticles for Murine Triple Negative Breast Cancer Treatment**

*Amirhosein Kefayat ^1^, Maryam Hosseini ^2^, Fatemeh Ghahremani ^3^,* *Nafise Arbab Jolfaie ^4^, Mohammad Rafienia^, 4^**

^1^ Department of Oncology, Isfahan University of Medical Sciences, Isfahan 81746-73461, Iran.

^2^ Department of Chemistry, Amirkabir University of Technology (Tehran Polytechnic), Tehran 1591634311, Iran.

^3^ Department of Medical Physics and Radiotherapy, Arak School of Paramedicine, Arak University of Medical Sciences, Isfahan, Iran.

^4^ Biosensor Research Center, Isfahan University of Medical Sciences, Isfahan, Iran

**Corresponding author** ***:**

Mohammad Rafienia

Tel: +983137922480

Postal code: 81746-73461

E-mail: m_rafienia@med.mui.ac.ir

**Running title:** CS-FA-MBZ nanoparticles made implants for breast cancer treatment


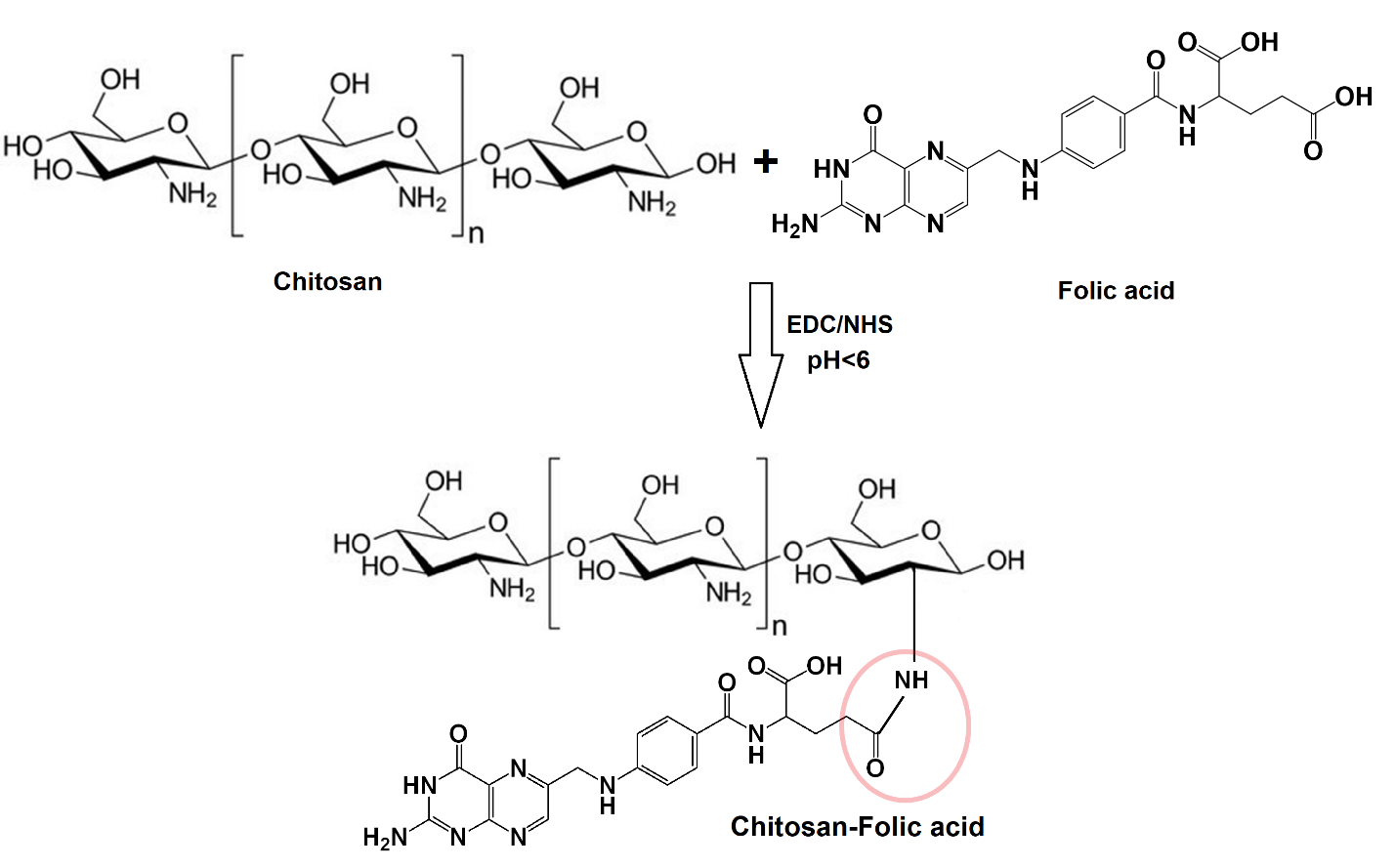


**Figure S1:** Schematic illustration of folic acid conjugation to chitosan in presence of EDC as carboxyl activating agent to produce CS-FA.


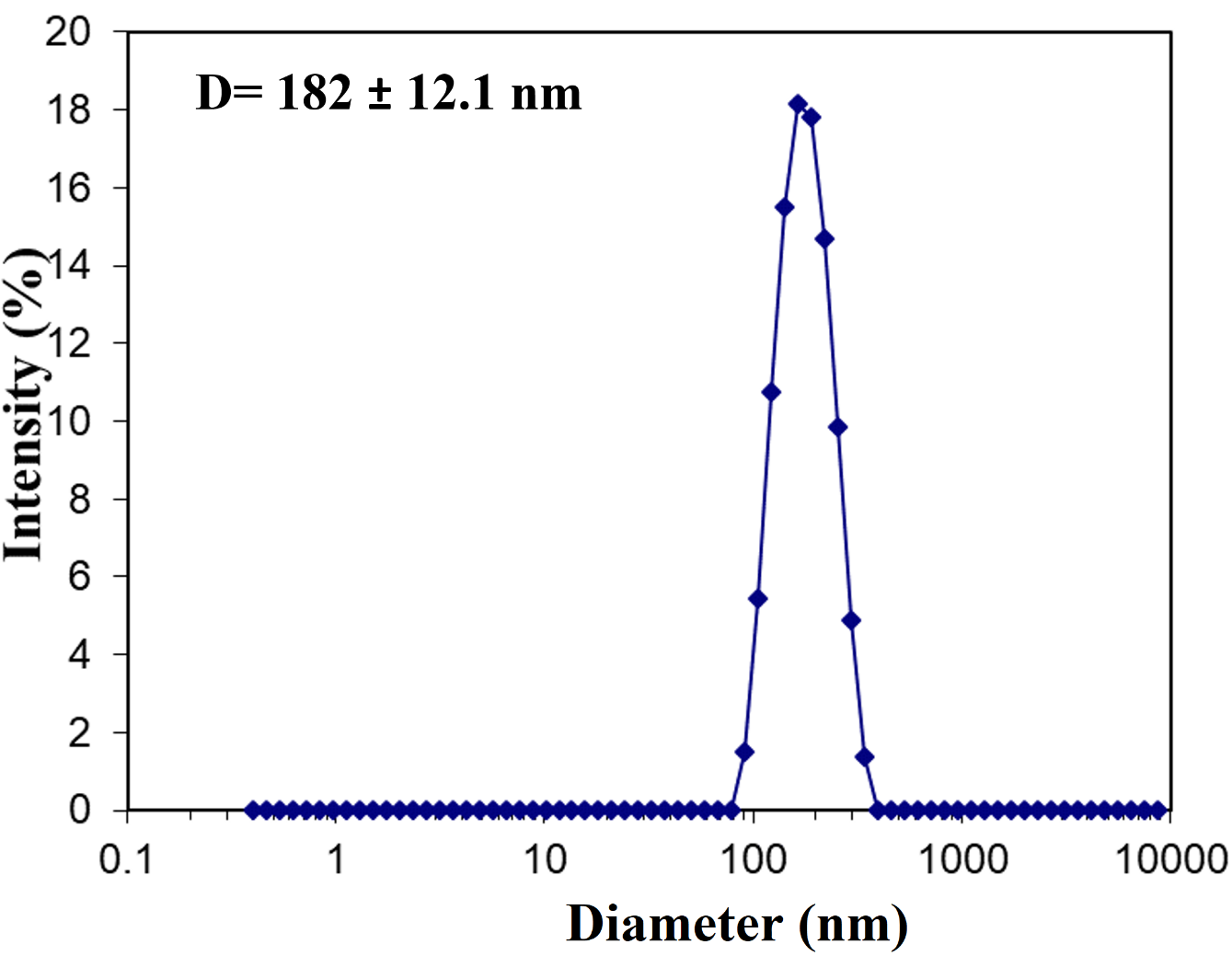


**Figure S2:** The CS-FA-MBZ nanoparticles hydrodynamic size distribution according to DLS measurements.


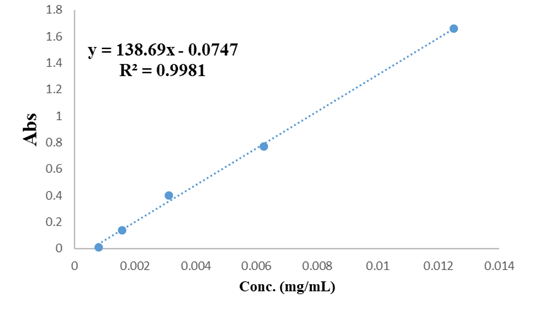


**Figure S3**: Standard calibration curve of MBZ.

| **Table S1.** General appearance and behavioral observations for Control and CS-Fa-MBZ groups (n=5). | | |
| --- | --- | --- |
| **Parameters** | **Control** | **CS-FA-MBZ** |
| Food/Water refuse | Absent | Absent |
| Aggressiveness | Absent | Absent |
| Eye and ears | Normal | Normal |
| Convulsion | Absent | Absent |
| Salivation | Absent | Absent |
| Diarrhea | Absent | Absent |
| Breathing | Normal | Normal |
| Heart beat | Normal | Normal |
| Activity | Active | Active |
| Anorexia | Absent | Absent |
| Cachexia | Absent | Absent |
| Mortality | Absent | Absent |
